# Heme oxygenase-1 and neopterin plasma/serum levels and their role in diagnosing active and latent TB among HIV/TB co-infected patients: a cross sectional study

**DOI:** 10.1186/s12879-021-06370-7

**Published:** 2021-07-27

**Authors:** Esther Uwimaana, Bernard S. Bagaya, Barbara Castelnuovo, David P. Kateete, Anguzu Godwin, Noah Kiwanuka, Christopher C. Whalen, Moses L. Joloba

**Affiliations:** 1 Department of Immunology & Molecular Biology, School of Biomedical Sciences, Makerere University College of Health Sciences, Kampala, Uganda; 2 Immunology Laboratory, School of Biomedical Sciences, Makerere University, Kampala, Uganda; 3 Infectious Diseases Institute, Makerere University College of Health Sciences, Kampala, Uganda; 4 School of Public Health, Makerere University College of Health Sciences, Kampala, Uganda; 5 Epidemiology & Biostatistics, Global Health Institute, University of Georgia Athens, Athens, USA

**Keywords:** Latent tuberculosis infection, Active tuberculosis, Biomarker

## Abstract

**Background:**

Tuberculosis (TB) diagnosis in the context of HIV co-infection remains challenging. Heme oxygenase 1 (HO-1) and neopterin have been validated as potential biomarkers for TB diagnosis. Latent TB infection (LTBI) is diagnosed using tuberculin skin test (TST) and interferon gamma release assays (T-Spot and QuantiFERON TB gold tests, respectively). However, these tests have shown challenges and yet diagnosing LTBI is important for the overall control of TB. This study was conducted to determine the levels of H0–1 and neopterin, and their role in the diagnosis of TB among individuals enrolled in the Community Health and Social Network of Tuberculosis (COHSONET) study and the Kampala TB Drug Resistance Survey (KDRS).

**Methods:**

This was a nested cross-sectional study. Plasma and serum samples collected from 140 patients at Mulago National Referral Hospital, Kampala Uganda were used. *M.tb* culture was performed on sputum to confirm active TB(ATB) and QuantiFERON TB gold test to confirm latent TB infection (LTBI). ELISAs were performed to determine the levels of HO-1 and neopterin. Data analysis was done using t-test and Receiver Operating Characteristic curves to determine the diagnostic accuracy.

**Results:**

HO-1 levels among active tuberculosis (ATB)/HIV-infected patients and LTBI/HIV-infected patients were 10.7 ng/ml (IQR: 7.3–12.7 ng/ml) and 7.5 ng/ml (IQR: 5.4–14.1 ng/ml) respectively. Neopterin levels among ATB/HIV-positive patients and LTBI/HIV-positive patients were 11.7 ng/ml (IQR: 5.2.4 ng/ml) and 8.8 ng/ml (IQR: 2.4–19.8 ng/ml), respectively. HO-1 showed a sensitivity of 58.57% and a specificity of 67.14% with area under the curve (AUC) of 0.57 when used to discriminate between ATB and LTB. Neopterin showed an AUC of 0.62 with a sensitivity of 57.14% and a specificity of 60.0% when used to distinguish ATB from LTB.

**Conclusion:**

There was no in significant difference in HO-1 concentration levels of ATB individuals compared to LTB individuals. There was a significant difference in neopterin concentrations levels of ATB individuals compared to latently infected individuals. Findings from this study, show that HO-1 and neopterin have poor ability to distinguish between ATB and LTB.

**Supplementary Information:**

The online version contains supplementary material available at 10.1186/s12879-021-06370-7.

## Background

In 2016, more than 2 billion people globally were estimated to be latently infected with *Mycobacterium tuberculosis (M. tb)*. In 2017, 10 million people fell ill with tuberculosis (TB), and 1.6 million died from the disease (including 0.3 million among people living with HIV). TB is the leading cause of death among HIV positive individuals globally [1]. In Uganda, the prevalence of latent tuberculosis infection (LTBI) was estimated to be 49% among adults [2, 3]. LTBI is diagnosed using the tuberculin skin test (TST) and interferon gamma release assays (T-Spot and QuantiFERON TB gold test). However, these diagnostic tests have shown challenges, for example, TST is associated with false positives or negatives especially among immunocompromised individuals like HIV/TB co-infected individuals with impaired cell-mediated immunity [4].

The QuantiFERON test is technology-intensive, requires expertise and difficult to implement in resource-limited settings. More so, all these tests cannot tell whether one has a current, cleared, progressed to active infection as they are based on infection with *M. tb* and give a positive result for both latently and actively infected patients. Hence the difficulty in distinguishing active tuberculosis (ATB) from LTBI during TB diagnosis [5].

As a result, biomarkers such as *M. tb* thymidylate kinase (TMKmt) antigen [6], Lipoarabinomannan(LAM) [7], heme oxygenase 1(HO-1 [8] and neopterin [9] have been studied. Studies have shown HO-1 and neopterin to successfully distinguish LTBI from ATB [8, 9]. HO-1 is a key stress response enzyme that is highly expressed in the lung tissue during *M. tb* infection and an anti-oxidant that degrades heme to iron, bilverdin, and carbonmoxide [10]. Neopterin is a product of guanosine triphosphate and is produced by human macrophages upon stimulation with the Th1 cell-derived cytokine interferon-gamma. Neopterin has been shown to be a marker of immune activation during M. tb infection [11].

There is no rapid diagnostic test to accurately distinguish LTBI from ATB. The sensitivity of TST is reduced for persons with impaired cell-mediated immunity as a reaction to tuberculin is impaired in individuals with HIV infection [12] and yet diagnosing LTBI is important for the overall control of TB.

More importantly, offering anti-TB treatment to individuals with LTBI significantly decreases their risk of developing ATB [13]. HIV infection increases the risk of reactivation of LTBI as infection with HIV is the most powerful known risk factor predisposing for *M. tb* infection [14] and progression to active disease, which increases the risk of latent TB reactivation.

A study done in India in 2013 on plasma HO-1 levels to distinguish ATB from LTBI showed that HO-1 plasma levels were elevated in those with ATB compared to individuals with LTBI and the healthy controls [8]. However, HO-1 levels in ATB and LTBI have not been studied in several other settings including sub-Saharan Africa. Another study in India, on serum neopterin levels in HIV infected patients with and without TB showed serum neopterin levels to be highest in HIV positive individuals with ATB and lowest in healthy controls [9]. We hypothesize that HO-1 and neopterin levels are elevated in individuals with *M. tb* infection. Therefore, this study aimed to determine the plasma levels of HO-1 and neopterin and their diagnostic accuracy in diagnosing TB among ATB/HIV-infected patients, and LTBI/HIV-infected patients compared to sputum culture and QuantiFERON TB gold test.

## Methods

### Study design and setting

This was a cross-sectional study conducted at the Immunology laboratory of the Department of Immunology and Molecular Biology, School of Biomedical Sciences, College of Health Sciences, Makerere University Kampala, Uganda. This study aimed to determine the plasma levels of HO-1 and neopterin, and their diagnostic accuracy in diagnosing TB among ATB/HIV-infected patients, and LTBI/HIV-infected patients compared to sputum culture and QuantiFERON TB gold test.

### Participants and samples

Two groups of samples were included: Samples from ATB/HIV-infected patients (pre-qualified by sputum culture and microscopy), LTB/HIV-infected individuals (pre-qualified by QuantiFERON-TB GOLD test).

Archived samples collected by the Community Health and Social Network of Tuberculosis (COHSONET) and Kampala TB Drug Resistance Survey (KDRS) studies were used in this study. The population size of the COHSONET and KDRS studies was 2279 and 473 participants, respectively. Consecutive sampling was used when retrieving the samples in this study, where all samples which met the inclusion criteria were enrolled into the study. These samples were stored at -80 °C.

**Fig. 1:**
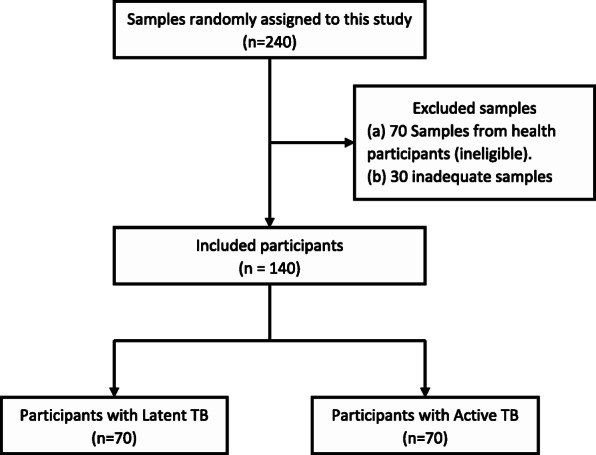
Flow chart of all excluded and included participants

Samples whose volume was inadequate to complete the laboratory tests and samples for patients who did not consent for future use of the samples were excluded (Fig. 1). De-identified data coded with participant identification numbers were extracted from the parent studies’ databases.

The sample size was calculated using a desired precision (0.06), sensitivity (91.8%) and specificity (94.9%) by Andrade and colleagues in 2013 [8]. The total estimated sample size for this study was 140 participants (70 patients with ATB/HIV-infected patients and 70 individuals with LTBI/HIV-infected.

### Laboratory procedures

HIV testing and laboratory diagnosis of both ATB and LTBI were previously determined by the COHSONET and KDRS studies. Briefly, HIV antibody testing was done in parallel using Abbot Determine (Abbott Laboratories Abbott Park IL, USA) and double-well run Vironostika HIV Uni-form II Ag/Ab (BioMerieux Boxtel, Netherlands). The Generic Biorad HIV-1/ HIV-2 plus O-(Enzyme-Linked Immunosorbent Assay) ELISA kit (Biorad Laboratories, Redmond WA, USA) was used as the tie-breaker. All tests were performed in accordance with the manufacturers’ instructions [15].

#### LTBI and ATB diagnosis

All samples used were designated as either LTBI or ATB using Quantiferon gold IGRA assay [16] and acid fast bacillus (AFB) smear microscopy/culture, respectively [15].

### Measurement of plasma HO-1 and neopterin

HO-1 and neopterin levels in the samples were determined using the human HO-1 ELISA kits (Xpress Biotech International) and human neopterin ELISA kits (Express Biotech International) respectively after optimizing by running different sample dilutions.

### HO-1 ELISA

Sandwich ELISA was used as the method of choice. The anti-HO-1 antibody was pre-coated onto the plate and the biotin-conjugated anti-HO-1 antibody was used as the detection antibodies. The standards, test samples, and detection antibody were added to wells subsequently and washed with wash buffer. Horseradish Peroxidase (HRP)-streptavidin was added, and unbound conjugates were washed away with wash buffer. 3,3′,5,5′-Tetramethylbenzidine (TMB) substrates were added followed by a stop solution. The colour change was determined spectrophotometrically at a wavelength of 450 nm. The concentration of HO-1 in the sample was determined by comparing the optical density (OD) of samples to the standard curves.

To determine the level of HO-1 enzyme in the samples, a sandwich ELISA was done using commercially obtained kits (Express Biotech International Cat No. XPEH3234). HO-1 kits and the samples were removed from the refrigerator and allowed to attain room temperature. The standard solution was serially diluted to obtain different dilutions. The HO-1 ELISA protocol was optimized by first running known samples before running the test samples. The plated wells were washed using 350 μl phosphate-buffered saline (PBS) in each well two times with a soaking time of 1 min and then blotted to dry. 0.1 ml of 20 ng/ml, 10 ng/ml, 5 ng/ml, 2.5 ng/ml, 1.25 ng/ml, 0.625 ng/ml, 0.313 ng/ml standard solutions were aliquoted into the standard wells. 0.1 ml of the sample (in duplicate) was aliquoted into the sample wells. The plate was sealed with a cover and incubated at 37 °C for 90 min. The cover was removed, and contents discarded then washed plate two times using wash buffer. 0.1 ml Biotin-detection antibody working solution was added into the above wells and plate incubated at 37 °C for 60 min. The cover was removed and washed three times with wash buffer.

Following washing, 0.1 ml of Streptavidin-Biotin Complex (SABC) working solution into each well, covered and incubated at 37 °C for 30 min. The plate was washed five times with wash buffer. Then 90 μl of TMB substrate was added into each well, the plate covered and incubated at 37 °C in the dark for 20 min. Then 50 μl of stop solution was added into each well and mixed thoroughly and the colour changed from blue to yellow immediately. The plate was read at 450 nm.

### Neopterin ELISA

Competitive ELISA was used as the method. The microtiter plate was pre-coated with neopterin. During the reaction, neopterin in the sample or standard competes with a fixed amount of neopterin on the solid phase supporter for sites on the Biotinylated Detection Antibody specific to neopterin. Excess conjugate and unbound sample or standard were washed from the plate and HRP-Streptavidin was added to each well and incubated. Then TMB substrate was added to each well followed by a stop solution. The color change was determined spectrophotometrically at a wavelength of 450 nm. The concentration of neopterin in the sample was determined by comparing the optical density of samples to the standard curves.

To determine the concentration levels of neopterin in the samples, a competitive ELISA was performed using a commercially prepared neopterin kit (Cat No. XPEH3413). Neopterin kits and the samples were removed from the refrigerator and allowed to attain room temperature. The standard solution was serial diluted and the protocol optimized. The plate was washed two times before adding standard, sample, and controls. Then 50 μl of sample and standard solution were added to the wells. Immediately 50 μl of Biotin-detection antibody was added to each well and incubated for 45 min at 37 °C. The plate was then washed three times using phosphate buffer. 100 μl SABC working solution was added to each well and incubated for 30 min at 37 °C, followed by washing the plate five times. 90 μl of TMB substrate was added to each well and incubated for 20 min at 37 °C. Followed by 50 μl stop solution was added to the wells. The plate was read at 450 nm.

### Quality assurance and control

Sample locations on the plate were mapped using ELISA worksheets during the assay. All samples and standards were run in duplicates. Blanks were run during all the assays to control for background reading. All reagents were properly thawed before use and raw data was entered and double-checked.

### Data management and analysis

The Optical densities (ODs) generated from the ELISA reader were entered into Microsoft excel. Data was cleaned by subtracting ODs of the blanks (background) from those of test wells. Standards were used to draw a standard reference curve from which the sample ODs were converted into HO-1 and neopterin concentrations in ng/ml (provided in supplementary material). Median values with interquartile ranges (IQR) were used as measures of central tendency. HO-1 and neopterin levels were compared among the study groups using the t-test. Receiver Operator Characteristics (ROC) curves were designed to test the diagnostic accuracy of HO-1 and neopterin. The statistical analysis was done using Graphpad Prism version 8.1.

## Results

### Participant baseline characteristics

A total of 140 archived participants’ plasma/serum samples were included in this study. The participants were stratified into two groups; 70(50%) of the participants were ATB/HIV-infected patients and 70 (50%) were LTB/HIV-infected individuals. Eighty one (57.8%) of the participants were male and the median age of the study participants was 26 years (IQR: 22-34 years). Details of participant demographic characteristics stratified by the study group are summarized in Table 1.

**Table 1:**
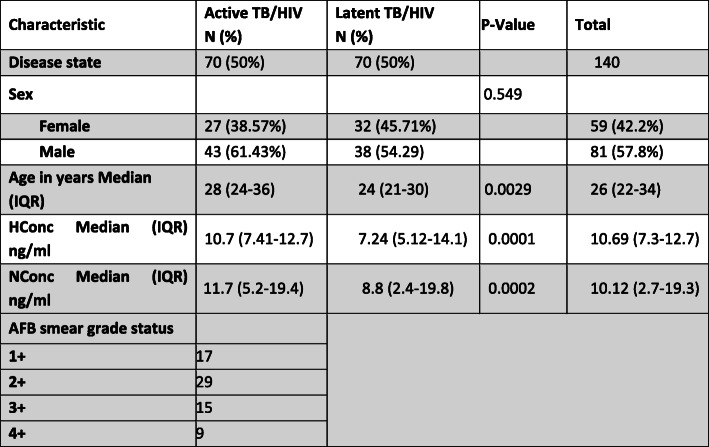
Patient baseline characteristics stratified by TB status

*IQR* Interquartile range; Hconc median, median concentration levels of heme oxygenase 1; NConc median, median concentration levels of neopterin

### HO-1 levels in serum/plasma

**Fig. 2:**
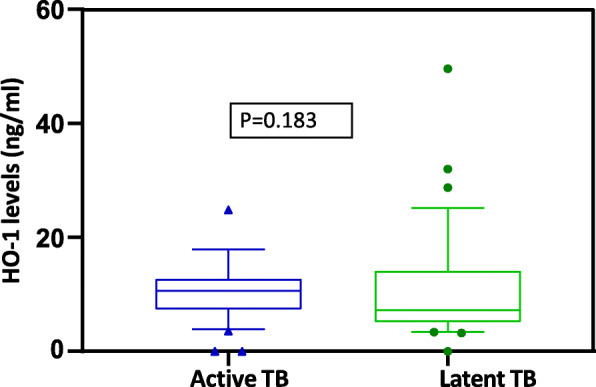
Concentration HO-1 levels in plasma/serum among active TB patients and latent TB patients. The boxes show median and interquartile ranges, whiskers show the 5th and 95thpercentiles, dots represent outliers, Asterisk (*) indicate significant results (*P* < 0.05)

The median concentration levels of HO-1 among ATB/HIV-infected patients and LTBI/HIV-infected individuals, were 10.7 ng/ml (IQR: 7.3–12.7 ng/ml) and 7.5 ng/ml (IQR: 5.4–14.1 ng/ml) respectively (See Table 1). We compared HO-1 levels among the two study groups and found no significant difference in HO-1 levels among ATBI/HIV-infected patients compared to LTBI/HIV-infected individuals (*P*-value = 0.183) (Fig. 2).

### Neopterin levels in serum/plasma

The median concentration levels of neopterin among ATB/HIV-infected patients and LTBI/HIV-infected individuals, were 11.7 ng/ml (IQR: 5.2–19.4 ng/ml) and 8.8 ng/ml (IQR: 2.4–19.8 ng/ml) respectively (See Table 1).

**Fig. 3:**
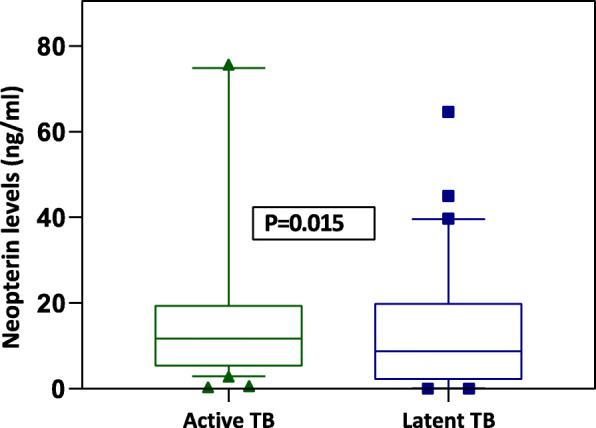
Concentration of neopterin levels(ng/ml) among active TB patients and latent TB patients. The boxes show median and interquartile ranges, whiskers show the 5^th^ and 95^th^percentiles, dots represent outliers, Asterisk (*) indicate significant results (*P* < 0.05)

There was a significant difference in the concentration levels of neopterin among ATBI/HIV-infected patients compared to LTBI/HIV-infected individuals (*P*-value = 0.015) (Fig. 3).

### Diagnostic accuracy of HO-1

**Fig. 4:**
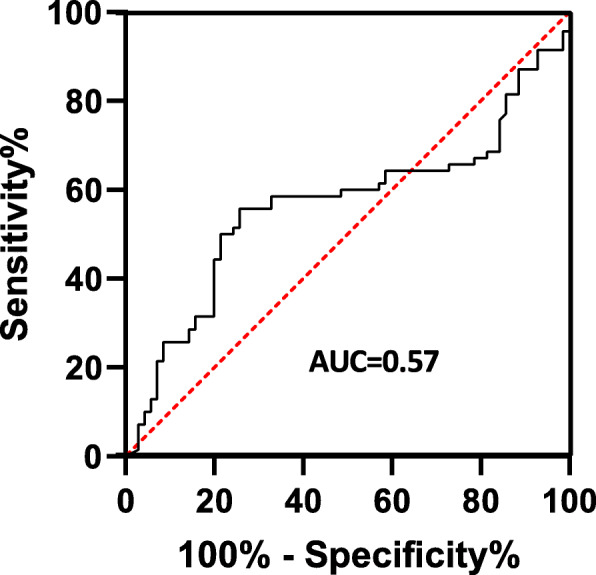
Receiver operating characteristic curve for HO-1 as a diagnostic biomarker for ATB and LTB

To explore the possibility of using HO-1 as a possible diagnostic biomarker for ATB and LTBI, receiver operating characteristics (ROC) curves were used to assess the diagnostic accuracy of HO-1 in the diagnosis of TB. In testing HO-1 as a marker of ATB and Latent TB, HO-1 showed an AUC of 0.57 (95% CI, 0.4664–0.6645) (Fig. 4). A highest diagnostic accuracy for HO-1 was obtained by using a cut off value of > 8.95 ng/ml, with a sensitivity of 58.57% (95% CI,46.88–69.37%) and a specificity of 67.14% (95% CI, 55.50 to 77.00%).

Negative predictive (NPV) and Positive Predictive Values (PPV) were also computed from the above sensitivity and specificity at a prevalence of TB of 50% (70/140). NPV was 63.6% and PPV was 62.2%.

### Diagnostic accuracy of neopterin

**Fig. 5:**
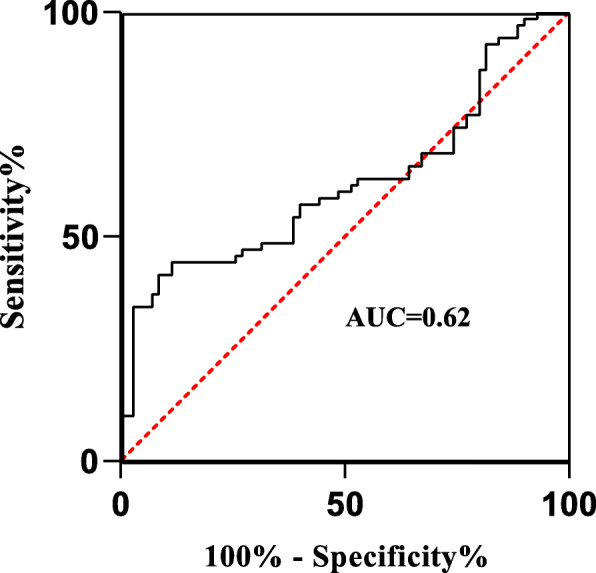
Receiver Operating Characteristic curve for neopterin as a diagnostic biomarker for ATB and LTB diagnosis

In exploring neopterin as a marker for ATB and LTB diagnosis, neopterin showed an AUC of 0.62 (95% CI, 0.5236–0.7135) and a diagnostic accuracy obtained using a cut-off > 10.12 ng/ml with a sensitivity of 57.14% (95% CI, 45.48 to 68.06%) and a specificity of 60.0% (95% CI, 48.29 to 70.67%). NPV and PPV were also computed from the above sensitivity and specificity at a prevalence of TB of 50% (70/140). NPV was 57% and PPV was 56.2% (Fig. 5).

## Discussion

In this study, HO-1 and neopterin concentration levels could not differentiate between individuals with ATB and latently infected individuals. Although we found a significant difference in the median serum/plasma concentration levels of neopterin (Fig. 3) between the two study groups, there was no significant difference in concentration levels of HO-1 (Fig. 2). While the current diagnostic methods cannot distinguish between ATB and LTB, previous studies have shown several biomarkers to discriminate ATB from LTB such as LAM [17], *M.tb* TMK antigen [6], neopterin, and C-reactive protein [18]. However, no study had been carried out to test the diagnostic potential of HO-1 and neopterin in a sub-Saharan population. Neopterin is synthesized by macrophages upon stimulation with cytokine interferon-gamma, and several studies have shown its concentrations to correlate with the extent and activity of disease, whereas HO-1 is an intracellular enzyme expressed in many cell types and tissues that is induced during cellular stress.

The findings of this study differ from those of a study by Michael Eyeshot et al.*,* (2016) which revealed that urine neopterin levels were significantly higher in ATB patients than in latently infected persons [18]. Another study on plasma HO-1 levels distinguished latently or successfully treated TB from active disease also showed that HO-1 levels were highest among patients with ATB compared to patients with LTBI and healthy controls [8]. These findings also differ from those obtained in this study as there was no significant difference in the concentration levels of HO-1.

Receiver operator characterization of HO-1 was done as a potential biomarker for ATB and LTB diagnosis. HO-1 failed to distinguish between individuals with active disease and those who were latently infected (Fig. 4).

A study carried out in Southern India demonstrated that HO-1 had the highest discriminatory power with a 23.5% higher specificity in distinguishing ATB from LTBI compared to SAA (94.9% vs. 71.4%, respectively) and 48.8% higher specificity compared to CRP (94.9% vs. 46.1%, respectively [8]. The difference in the diagnostic accuracy compared to our study could be attributed to the sample size difference in different study groups whereby the number of ATB individuals was much more than the latently infected group and the healthy donors. Receiver operator characterization of neopterin demonstrated neopterin to be a fair biomarker for ATB and LTB diagnosis (Fig. 5). Generally, Both HO-1 and neopterin could not discriminate ATB from LTB. Although other studies have showed these markers to be potential biomarkers that could be employed in TB diagnosis, our study did not find them useful in TB diagnosis as they failed to distinguish between ATB and LTB.

Limitation: HO-1 and neopterin expression are known to increase in patients with other pathologies, including other non –tuberculosis infections therefore, such a test may require to be optimized by combination with a secondary assay to provide an accurate diagnosis of *M. tb* infection.

## Conclusions

In summary, the findings show that HO-1 and neopterin have poor ability to distinguish between ATB and LTB in this population which differs from results from studies done in Asian populations. To better understand role of these biomarkers, these markers need to be further investigated and validated in other populations.

## Supplementary Information

**Additional file 1: Supplementary material.** Standards were used to draw a standard reference curve from which the sample ODs were converted into HO-1 and neopterin concentrations in ng/ml.

## Data Availability

The data that support the findings of this study are available from the parent studies, but restrictions apply to the availability of these data, which were used under license for the current study, and so are not publicly available. Data are however available from the authors upon reasonable request and with permission of parent studies.

## Abbreviations

ATP: Adenosine Triphosphate
AIDS: Acquired Immune Deficiency Syndrome
ATB: Active Tuberculosis
CI: Confidence Intervals
COHSONET: Community Health and Social Network of Tuberculosis
ELISA: Enzyme-Linked Immunosorbent Assay
Fig: Figure
Hconc: Concentration of Heme oxygenase 1
HIV: Human Immunodeficiency Virus
HO-1: Heme oxygenase 1
HRP: Horseradish Peroxidase
IGRA: Interferon-Gamma Release Assay
KDRS: Kampala Drug Resistance Survey
LTB: Latent tuberculosis
LTBI: Latent Tuberculosis Infection
*M.tb*: *Mycobacterium tuberculosis*
Nconc: concentration of neopterin
ODs: Optical Densities
SABC: Streptavidin-Biotin Complex
TB: Tuberculosis
TMB: 3,3′,5,5′-Tetramethylbenzidine
TST: Tuberculin Skin Test
WHO: World Health Organization
